# A Network Analysis Study Investigating Posttraumatic Stress Disorder and Dissociation Comorbidity in a UK Armed Forces Veteran Sample

**DOI:** 10.1192/j.eurpsy.2022.212

**Published:** 2022-09-01

**Authors:** C. Armour, E. Mcglinchey

**Affiliations:** Queen’s University Belfast, Psychology, Belfast, United Kingdom

**Keywords:** Network Analysis, comorbidity, PTSD, Dissociation

## Abstract

**Introduction:**

An established body of literature has identified that PTSD and dissociation are comorbid. Furthermore, the DSM introduced a dissociative subtype of PTSD into their most recent update; DSM-5.

**Objectives:**

The current study aimed to examine symptom-level associations between PTSD and dissociation using network analysis among UK Armed Forces veterans resident in Northern Ireland (NI) to identify if there are certain symptoms that may act as bridges between the two constructs.

**Methods:**

A large scale cross sectional survey was conducted examining the physical and mental wellbeing of UK Armed Forces Veterans living in NI. The total eligible sample size was 619 (89.8% male), with a mean age of 55.38 years (SD = 10.41). Two networks were estimated, (1) a network consisting of 20 DSM-5 PTSD items and (2) a network consisting of 20 PTSD items and four dissociative items. Expected influence bridge centrality was calculated to examine symptoms with the most/strongest cross-domain associations (i.e. between PTSD and dissociation). The presence of meaningful clustering among symptoms was also explored.

**Results:**

The PTSD symptoms *‘concentration problems’*, *‘flashbacks’* and *‘negative emotional state’* had the highest relative bridge expected influence centrality. Of the four dissociative items, *‘gaps in awareness’* had the highest relative bridge expected influence centrality, followed by *‘cognitive-behavioural re-experiencing’.* A community structure of five clusters was detected. Four clusters reflected each subscale of the PCL-5 PTSD items and the final cluster reflected the dissociation items.

**Conclusions:**

This study extends our understanding of PTSD and disociation comorbidity by investigating symptom level relationships; potentially informing future treatments and interventions.

**Disclosure:**

No significant relationships.

